# Anti-citrullinated protein antibodies in the diagnosis of rheumatoid arthritis **(**RA): diagnostic performance of automated anti-CCP-2 and anti-CCP-3 antibodies assays

**DOI:** 10.1007/s10067-017-3684-8

**Published:** 2017-06-03

**Authors:** Ine Vos, Christof Van Mol, Leendert A. Trouw, Michael Mahler, Jaap A. Bakker, Jan Van Offel, Luc De Clerck, Tom W. Huizinga

**Affiliations:** 1 Department of Rheumatology, Leiden University Medical Center, Leiden, the Netherlands; 2 Department of Rheumatology, University Hospital of Antwerp, Edegem, Belgium; 3 Netherlands Interdisciplinary Demographic Institute/KNAW/UG, The Hague, the Netherlands; 4 INOVA diagnostics, San Diego, CA USA; 5 Department of Clinical Chemistry and Laboratory Medicine, Leiden University Medical Center, Leiden, the Netherlands

**Keywords:** Diagnostic performance, Rheumatoid arthritis, Second generation anti-cyclic citrullinated peptide antibody, Third generation anti-cyclic citrullinated peptide antibody

## Abstract

This study compares the diagnostic performance of a second generation anti-cyclic citrullinated peptide antibody (CCP2) with a third generation anti-CCP antibodies assay (CCP3), as well as the combination of both tests. Serum samples of 127 patients were analyzed. IgG anti-CCP 2 and IgM rheumatoid factor were determined by EliA™ technique on a Phadia 250 instrument (Thermo Fisher Scientific), anti-CCP3 by the Quanta Flash™ anti-CCP3 IgG kit, BIO-FLASH Rapid Response Chemiluminscence Analyzer (INOVA Diagnostics). Diagnostic performance was compared using ROC-curves, sensitivity, specificity, likelihood ratios, and predictive values. Logistic regressions were used to investigate whether using both tests (anti-CCP2 and anti-CCP3) gives a better prediction of rheumatoid arthritis. At the manufacturer’s cut-offs sensitivity and specificity were 79.4 and 61.0% for CCP3 and 80.9 and 69.5% for CCP2. No significant differences could be observed regarding the areas under the curve (AUC) of both ROC-curves. The optimal cut-off point for CCP2 was 10.5 U/ml (sensitivity of 75.0% and specificity of 80.0%) and 5.6 U/ml for CCP3 (sensitivity of 86.9% and specificity of 61.0%). Binary logistic regressions indicated that the likelihood of having rheumatoid arthritis (RA) is significantly higher when testing positive on both CCP2 and CCP3 compared to CCP2 or CCP3 alone. In our cohort, comparable performance was found between the two CCP assays. Positivity for both CCP2 and CCP3 resulted in the most specific identification of RA patients. In patients with joint complaints suspected of having RA and with a weakly positive CCP 2 (≥7 and ≤16 U/ml) CCP3 testing could be of additive value for diagnosing RA.

## Introduction/objectives

Rheumatoid arthritis (RA) is a heterogeneous condition. This is well illustrated by the highly variable course the disease may follow in different individuals. RA is characterized by inflammation and ultimately damage of the joints. Modern treatment, therefore, is based on aggressive anti-rheumatic therapy in the early phase of the disease and consequently delays the disease progression [1]. Although the diagnosis of RA is mainly made on clinical grounds, disease-specific markers are of additional value for an increasing proportion of the RA patients [2]. Historically, rheumatoid factor (RF) was the main element in the serological diagnosis of RA and the only laboratory diagnostic parameter included in the 1987 American College of Rheumatology (ACR) criteria for the classification of RA [3]. However, RF is not very specific for this disease and can also be detected in patients with other rheumatic disorders, infections, as well as in apparently healthy individuals [4]. The other tests available at that time were anti-perinuclear factor and anti-keratin antibody. However, their diagnostic utilities were initially limited due to low sensitivity, specificity, and methodology [5].

In 1998, Schellekens et al. [6] introduced an ELISA based on these original findings now using citrullinated peptides (CCP). The first generation of anti-CCP antibodies (anti-CCP1) test revealed a higher specificity for RA in comparison to the RF test [7]. At the end of 2002, second generation anti-CCP antibodies tests were developed, with different cyclic peptides and improved performance characteristics showing an even better specificity for RA [7]. Following anti-CCP2, a third generation anti-CCP test (anti-CCP3) has been developed to increase the sensitivity for the detection of patients with RA. In sum, both sensitivity and specificity of the anti-CCP tests are significantly higher than those of the RF test [8].

Several scholars compared the diagnostic performance of anti-CCP2 and anti-CCP3, but conflicting evidence emerges from these studies. On the one hand, several studies conclude that the anti-CCP3 test has no apparent diagnostic advantage compared with the anti-CCP2 test [8–16]. On the other hand, other studies showed a higher sensitivity of the anti-CCP3 compared to anti-CCP2 tests [7, 16–18].

Recently, it has been speculated that the reported higher sensitivity of CCP3 may only be found in cohorts with early RA, whereas the sensitivity may be similar in groups with established disease [2, 19]. A study performed by Jaskowski et al. [20] found no statistically significant difference in sensitivity and specificity between anti-CCP3 and anti-CCP2. Instead, they showed that anti-CCP3 antibodies were more prevalent than anti-CCP2 antibodies in RF-negative RA patients. These results were confirmed by a study performed by Swart et al. [19] comparing two anti-CCP tests using a routine patient cohort. They found that discrimination between RA and non-RA patients was better using CCP3. The most pronounced difference between CCP2 and CCP3, however, was found in RF-negative patients with a disease duration of ≤5 years. In this cohort (*n* = 31), the sensitivity of CCP3 was 51.6% compared to 38.7% for CCP2.

In sum, although the majority of studies comparing CCP2 and CCP3 detected no advantage of using CCP3 over CCP2, a few studies showed a higher sensitivity of the anti-CCP3 peptide assay compared to anti-CCP2 tests. Now, the CCP3 test has been developed for analysis on an automated analyzer, the BIO-FLASH instrument, and this assay may have different properties as compared to ELISA-based assays. Therefore, here, we analyzed the differences between anti-CCP2 and anti-CCP3 on the BIO-FLASH in a setting of a routine patient population.

## Methods

### Study population

Samples were obtained from 127 consecutive patients for whom anti-CCP and IgM RF determination had been routinely ordered for investigation of joint disease between March 2013 and March 2014. Clinical data were retrospectively collected between February and August 2015 by reviewing the electronic medical records. Of the patients for whom CCP was ordered, we assessed both the anti-CCP2 assay and the anti-CCP3 assay. On a total of 127 patients, 68 patients were diagnosed with RA according to the revised 1987 ACR diagnostic criteria, 59 were classified as non-RA. The RA group was subdivided in patients with a disease duration of less than 2 years (*n* = 26), between 2 and 5 years (*n* = 18), and more than 5 years (*n* = 25). Furthermore, we divided the RA group according to the RF status resulting in a group of RF-positive patients (*n* = 47) and a group RF-negative patients (*n* = 15). The non-RA group consists of patients with osteoarthritis (*n* = 11), undifferentiated arthritis (*n* = 10), crystal arthropathy (*n* = 4), psoriatic arthritis (*n* = 4), and other diagnoses (*n* = 30). This last group consists of nine patients with other inflammatory rheumatic diseases (SLE *n* = 1, reactive arthritis *n* = 2, spondylartropathy *n* = 2, systemic sclerosis *n* = 1, amyloidosis *n* = 1, and mixed connective tissue disease *n* = 2) and 22 without clinical signs of an inflammatory rheumatic disease. Noteworthy, the group of undifferentiated arthritis consists of patients with arthritis not fulfilling the 1987 ACR criteria for RA up to 16 months of follow-up.

### Immunoassays

IgG anti-CCP2 antibodies and IgM RF were determined using the EliA™ technique (POhadia 250; Thermo Fisher Scientific, Uppsala, Sweden). Anti-CCP3 antibodies were determined using the Quanta Flash™ anti-CCP3 IgG kit (BIO-FLASH Rapid Response Chemiluminscence Analyzer; INOVA Diagnostics; San Diego, CA, USA). All assays were analyzed at the central clinical chemistry laboratory of the Leiden University Medical Centre.

### Statistical analysis

In order to compare the diagnostic performance of the tests, ROC analysis was performed. Furthermore, the areas under the curve (AUC) of both tests were compared to investigate whether one of the tests yielded significant advantages over the other. We calculated alternative cut-off points by the Youden’s index. Binary logistic regressions were used to investigate whether there is an advantage in diagnosing RA when using both tests compared to using a single CCP2 or CCP3 test. All analyses were performed with Stata 14 software.

## Results

### Prevalence of anti-CCP2, anti-CCP3, and RF in different disease cohorts

At the manufacturer’s cut-off point, 55/68 (80.9%) RA patients were positive for anti-CCP2, 53/68 (78.0%) for anti-CCP3 antibodies, and 45/68 (75.0%) for RF. In the control group, 18/59 (30.5%) were positive for anti-CCP2, 23/59 (39.0%) for anti-CCP3, and 19/57 (33.3%) for RF (Table 1). In the RF-negative RA group (*n* = 15), 8/15 (53.3%) were positive for CCP2, and 9/15 (60.0%) were positive for CCP3. In the group of RA patients with a disease duration less than 2 years, 20/26 (76.9%) were positive for anti-CCP2 as well as for anti-CCP3. In the group of patients with disease duration between 2 and 5 years, 13/18 (72.2%) were positive for anti-CCP2 positive and 14/18 (77.8%) for anti-CCP3. In patients with disease duration more than 5 years, 22/25 (88.0%) were CCP2 positive, and 20/25 (80.0%) were CCP3 positive.

**Table 1:** Prevalence of anti-CCP2, anti-CCP3, and rheumatoid factor in different disease cohorts

Disease	*N*	CCP2, no. pos (% pos)	CCP3, no. pos (% pos)	RF, no. pos (% pos)
RA	68	55 (80.9)	53 (78.0)	45 (75.0)
Disease duration				
<2 years	26	20 (76.9)	20 (76.9)	18 (69.2)
≥2 and <5 years	18	13 (72.2)	14 (77.9)	10 (55.5)
≥5 years	25	22 (88.0)	20 (80.0)	20 (80.0)
RF status				
RF negative	15	8 (53.3)	9 (60.0)	
RF positive	47	41 (87.2)	38 (80.9)	
Unknown	6	5 (83.3)	6 (100)	
Non-RA	59	18 (30.5)	23 (39.0)	19 (33.3)
Osteoarthritis	11	1 (9.1)	4 (36.4)	1 (9.1)
Undifferentiated arthritis	10	5 (50.0)	7 (70.0)	5 (50.0)
Crystal arthropathy	4	1 (25.0)	1 (25.0)	1 (25.0)
Psoriatic arthritis	4	0 (0.0)	0 (0.0)	2 (50.0)
Others	30	11 (36.6)	10 (33.3)	9 (30.1)

### Sensitivity and specificity/likelihood ratios/predictive values/area under the curve values obtained from ROC analysis

**Fig. 1:**
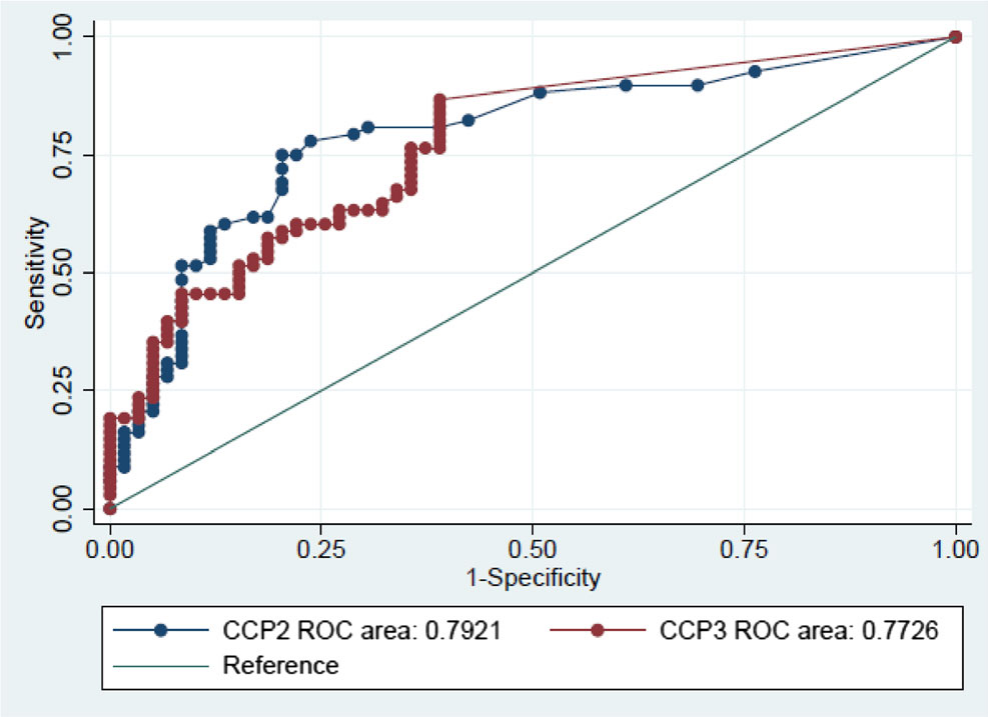
Comparative receiver operating characteristic (ROC) analysis for CCP2 and CCP3. The ROC-curves for both tests were comparable as shown by the area under the curve (AUC) values

In order to compare the diagnostic performance of the tests, ROC analysis was performed for CCP2 and CCP3 tests (Fig. 1). Using the manufacturers cut-off value (for CCP 2 < 7 U/ml and for CCP3 < 20 U/ml), the diagnostic sensitivity for anti-CCP2 and anti-CCP3 was 80.9 and 79.4%, respectively, while the specificity was 69.5 and 61.0%, respectively. The AUC values were 0.79 (95% CI: 0.71–0.87) for anti-CCP2 and 0.77 (95% CI: 0.69–0.85) for anti-CCP3. The LR+/LR− were 2.65/0.28 for anti-CCP2 and 2.04/0.34 for anti-CCP 3. In addition, the positive predictive value for CCP2 and CCP3 were respectively 75.3 (95% CI: 63.9–84.7) and 69.7% (95% CI: 58.1–79.8), and the negative predictive value 75.9% (95% CI: 62.4–86.5) for CCP2 and 70.6% (95% CI: 56.2–82.5) for CCP3. When comparing the AUC of both ROC-curves, no significant differences can be observed (*X* ^2^ = 0.41, *p =* 0.52). This result suggests there is no advantage of using one over the other test. Furthermore, we calculated alternative cut-off points by the Youden’s index. The optimal cut-off point for CCP2 was 10.5 U/ml with a sensitivity of 75.0% and specificity of 80.0% and 5.6 U/ml for CCP3 reflecting a sensitivity of 87.0% and specificity of 61.0%.

### Binary logistic regressions

As there is apparently no advantage of using one over the other test, it is worthwhile to explore whether a combination of the two tests is advantageous for diagnosing RA. Therefore, as a final analytical step, we ran binary logistic regressions with RA and non-RA as our dependent variable, controlling for gender and age. As can be observed in Table 2, model I indicates that patients scoring positively on CCP2 tests have 8.71 higher odds of having RA. Controlling for gender and age (both non-significant), 19% of the variation in outcome of RA is explained. Furthermore, model II shows that patients testing positive on CCP3 have 5.86 higher odds of having RA. CCP3 explains 14% of variation. In model III, both positivity of CCP2 and CCP3 are included. Both are significantly correlated with having RA, and the variation amounts to 22%. As a result, it is worthwhile to investigate which combination of CCP2 and CCP3 values yields higher probability of having RA.

**Table 2:** Binary logistic regression on the diagnosis of RA (odds ratios, standard error between brackets) (*n* = 127)

	Model I CCP2		Model II CCP3		Model III CCP2 and CCP3	
	OR (SE)	95%CI	OR (SE)	95%CI	OR (SE)	95%CI
Constant	0.25 (.230)	0.04–1.49	0.20 (.182)	0.04–1.17	0.16 (.147)*	0.03–0.97
CCP2 positivity	8.71 (3.64)***	3.84–19.77			5.56 (2.56)***	2.25–13.72
CCP3 positivity			5.86 (2.37)***	2.66–12.95	2.73 (1.27)*	1.10–6.78
Controls						
Age	1.01 (.014)	0.99–1.04	1.02 (.013)	0.99–1.04	1.01 (.014)	0.99–1.04
Gender	0.60 (.278)	0.25–1.49	0.75 (.330)	0.32–1.78	0.66 (.311)	0.26–1.66
*R* ^2^	.19		.14		.22	

*p < . 05, **p < .01, ***p < .001

**Fig. 2:**
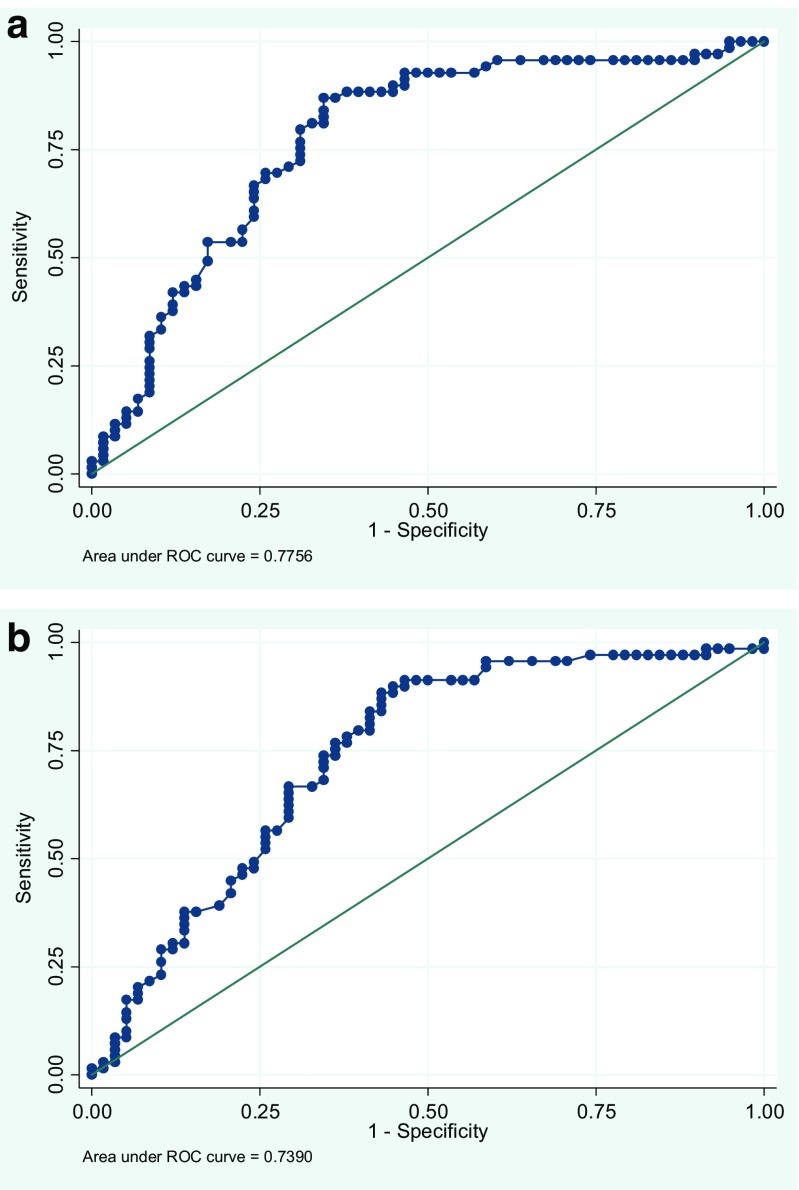
**a** Post-estimation of ROC-curve after logistic regression analysis, CCP2. **b** Post-estimation of ROC-curve after logistic regression analysis, CCP3

**Fig. 3:**
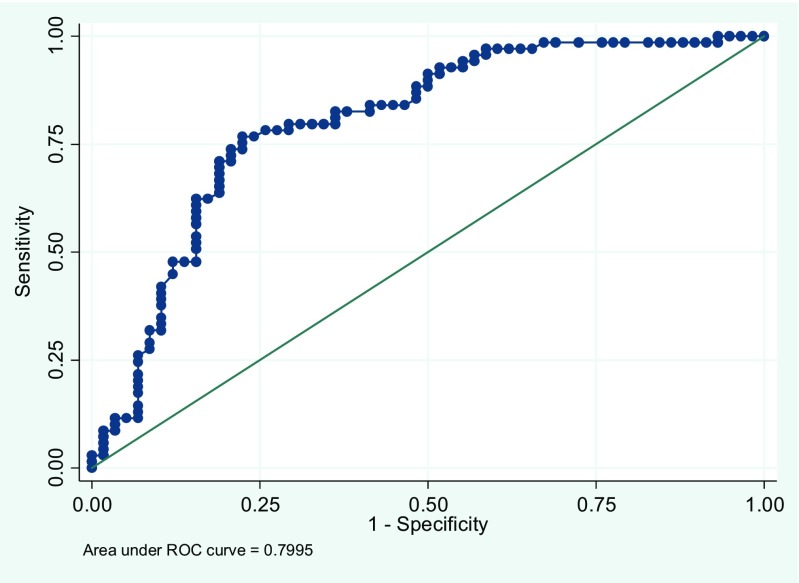
Post-estimation of ROC-curve after logistic regression analysis, CCP2, and CCP3

The logistic regression models were followed up by ROC-curves. As can be observed in Fig. 2a, b, the AUC values were 0.776 for anti-CCP2, and 0.739 for anti-CCP3. Combining CCP2 and CCP3 yields an area under curve value of 0.800, indicating an acceptable discriminating power of the model (Fig. 3).

Finally, in order to investigate which combination of CCP2 and CCP3 values leads to a higher probability of having RA, predicted probabilities of the combination of CCP2 and CCP3 values were calculated. In this analysis, we particularly focus on the weakly positive values of CCP2 (≥7 and ≤10 U/ml). As can be observed from Table 3, combining these weakly positive values of CCP2 with CCP3 can lead to a significant better prediction of having RA, compared to using the values of CCP2 alone.

**Table 3:** Combination of CCP2 and CCP3 values (U/ml), predicted probabilities of displaying RA (in percentages)

		CCP3 values													
CCP2 values	PPV CCP2	57.4	61	63.7	66	69	70.5	74.4	76.2	191	339.6	501	736.2	972.4	2662.6
7	44.9	45.0	45.3	45.5	45.6	45.9	46.0	46.3	46.4	55.2	66.0	76.0	86.7	93.1	100
8	45.2		45.4	45.6	45.8	46.0	46.1	46.4	46.5	55.3	66.1	76.1	86.7	93.1	100
9	45.5		45.6	45.7	45.9	46.1	46.3	46.6	46.7	55.4	66.2	76.2	86.8	93.1	100
10	45.8			45.9	46.0	46.3	46.4	46.7	46.8	55.6	66.3	76.3	86.9	93.2	100
11	46.1				46.2	46.4	46.5	46.8	47.0	55.7	66.4	76.4	86.9	93.2	100
12	46.4					46.5	46.7	47.0	47.1	55.8	66.6	76.5	87.0	93.2	100
13	46.7						46.8	47.1	47.2	56.0	66.7	76.6	87.0	93.3	100
14	47.0							47.2	47.4	56.1	66.8	76.7	87.1	93.3	100
15	47.2							47.4	47.5	56.2	66.9	76.8	87.2	93.3	100
16	47.5								47.6	56.4	67.0	76.9	87.2	93.4	100

## Discussion

Anti-CCP antibodies are known to be an important serological marker in the diagnosis of RA [2, 10, 21]. As a result, since 1998 several tests have been developed for a more accurate identification of RA patients. Most recently, third generation anti-CCP tests have been developed to increase the sensitivity for the detection of patients with RA. However, the academic literature comparing anti-CCP2 and anti-CCP3 assays presents conflicting evidence [7–18]. In this paper, we aimed to advance current knowledge by investigating the diagnostic performance of anti-CCP2 and anti-CCP3 assays separately as well as in combination, in a setting of a routine patient population.

Our analysis reveals no significant differences between the AUC of both ROC-curves, indicating there is no advantage of using one over the other test. However, the results clearly indicate that a combination of both tests increases the likelihood of diagnosing RA. Furthermore, although we observed a trend of higher CCP3 in earlier and RF-negative RA, this difference was not statistically significant. It is plausible this finding is caused by the limited number of samples.

When we compare our results to previous studies, the specificity of both CCP tests is remarkably lower. This might be attributed to the study design which was based on a routine setting. Instead of comparing RA with a healthy non-RA group, we included patients who were often already suspected of having RA. However, we argue this population which is particularly interesting for investigating whether CCP3 could be of additive value for CCP2. Consequently, we tried to define a subgroup of patients—based on CCP2 values—where CCP3 can be used as additive diagnostic value in diagnosing RA. Our results clearly show that combining the weakly positive values of CCP2 with CCP3 can lead to a significant better prediction of having RA, compared to using CCP2 assay alone. These findings suggest that a prediction model for having RA based on CCP3 values, in populations of patients with joint complaints and weakly positive CCP2, could be of additive value for clinical practice.

## Conclusions

Our data demonstrate that anti-CCP2 and CCP3 show comparable performance characteristics in our patients’ population. Although this conclusion should be taken with caution because of the relatively small number of patients analyzed, it is in line with previous studies. Of note, the specificity was remarkably lower than reported in various studies which should be considered when using the CCP test in a cohort with a high number of related diseases. Positivity for both CCP2 and CCP3 resulted in the most specific identification of the RA patients. Interestingly, however, this study shows that for patients with joint complaints suspected of having RA and with a weakly positive CCP 2 (≥7 and ≤16 U/ml), CCP3 testing could be of additive value for diagnosing RA.
